# Two Years of SARS-CoV-2 Omicron Genomic Evolution in Brazil (2022–2024): Subvariant Tracking and Assessment of Regional Sequencing Efforts

**DOI:** 10.3390/v17010064

**Published:** 2025-01-04

**Authors:** Ueric José Borges de Souza, Fernando Rosado Spilki, Amilcar Tanuri, Paulo Michel Roehe, Fabrício Souza Campos

**Affiliations:** 1 Bioinformatics and Biotechnology Laboratory, Campus of Gurupi, Federal University of Tocantins, Gurupi 77410-570, Brazil; 2 Molecular Microbiology Laboratory, Feevale University, Novo Hamburgo 93525-075, Brazil; fernandors@feevale.br; 3 Laboratory of Genetics and Immunology of Viral Infections, Department of Virology, Paulo de Góes Institute of Microbiology, Federal University of Rio de Janeiro, Rio de Janeiro 21941-902, Brazil; atanuri1@gmail.com; 4 Virology Laboratory, Department of Microbiology, Immunology, and Parasitology, Institute of Basic Health Sciences, Federal University of Rio Grande do Sul, Porto Alegre 90050-170, Brazil; proehe@gmail.com

**Keywords:** SARS-CoV-2, genomic surveillance, omicron subvariants, COVID-19, lineage tracking, public health

## Abstract

SARS-CoV-2, the virus responsible for COVID-19, has undergone significant genetic evolution since its emergence in 2019. This study examines the genomic diversity of SARS-CoV-2 in Brazil after the worst phase of the pandemic, the wider adoption of routine vaccination, and the abolishment of other non-pharmacological preventive measures from July 2022 to July 2024 using 55,951 sequences retrieved from the GISAID database. The analysis focuses on the correlation between confirmed COVID-19 cases, sequencing efforts across Brazilian states, and the distribution and evolution of viral lineages. Our findings reveal significant regional disparities in genomic surveillance, with São Paulo and Rio de Janeiro recovering the largest number of genomes, while Tocantins and Amazonas showed higher sequencing rates relative to their reported case numbers, indicating proactive surveillance efforts. We identified 626 distinct SARS-CoV-2 lineages circulating in Brazil, with dominant subvariants shifting over time from BA.5 in 2022 to XBB and JN.1 in 2023–2024. The emergence of new subvariants in this new epidemiological scenario underscores the importance of ongoing genomic surveillance to track viral evolution and inform public health strategies, providing valuable information to update vaccines and implement other measures, such as lockdowns, mask usage, social distancing, health education, and self-testing.

## 1. Introduction

In December 2019, a novel beta coronavirus was first identified in Wuhan, China [1]. This virus, now known as SARS-CoV-2, rapidly spread across the globe, infecting over 770 million people and causing more than 7 million deaths worldwide by October 2024 [2]. On 5 May 2023, the World Health Organization (WHO) declared an end to the global health emergency of the coronavirus disease (COVID-19), signaling a transition to the post-pandemic phase after three years of profound disruption to daily life, economies, and healthcare systems [3]. However, it is crucial to emphasize that this declaration does not imply the complete eradication of COVID-19. The virus continues to circulate, with transmission rates and effects varying across regions.

SARS-CoV-2, like other coronaviruses, possesses a large single-stranded RNA genome of approximately 30,000 nucleotides, making it one of the largest known RNA virus genomes [4]. The replication of this extensive genome is primarily facilitated by the 3′-to-5′ exoribonuclease (ExoN) activity within the non-structural protein 14 (nsp14), in conjunction with the RNA-dependent RNA polymerase (RdRp, nsp12) [4,5,6]. As is typical for RNA viruses, coronaviruses mutate frequently, often over the course of months to years, enabling their evolutionary trajectories to be tracked and analyzed in real time [7]. The accumulation and dissemination of mutations within viral populations are key drivers of their rapid evolution. Natural selection plays a critical role, favoring advantageous mutations, such as the D614G substitution, which has significantly increased viral transmissibility by enhancing the interactions between the viral spike protein and the human angiotensin-converting enzyme 2 (ACE2) receptor [7,8,9]. Ongoing genomic surveillance of these mutations is essential for tracking the viral evolution, mapping its geographic spread, and, more importantly, identifying changes that may increase transmissibility or confer immune evasion capabilities.

The mutation rate of SARS-CoV-2 ranges from 1 × 10^−6^ to 2 × 10^−6^ mutations per nucleotide per replication cycle [7,10,11], consistent with the rates observed in other beta coronaviruses [12,13]. This mutation rate is notably lower than that of other RNA viruses, such as human immunodeficiency virus (HIV), which has a mutation rate of approximately 10^−4^ to 10^−6^ mutations per nucleotide per replication cycle [14]; hepatitis C virus (HCV), with a rate of about 10^−5^ to 10^−6^ mutations per nucleotide per replication cycle [15]; and influenza A virus, whose RNA-dependent RNA polymerase (RdRP) exhibits a mutation rate ranging from 2.0 × 10^−6^ to 2.0 × 10^−4^ mutations per nucleotide per replication cycle [16,17,18].

Since the beginning of the COVID-19 pandemic, numerous SARS-CoV-2 variants have emerged globally, each characterized by distinct genetic mutations that have influenced the virus’s transmission dynamics and overall impact. Notable variants include B.1.1.7 (Alpha), B.1.351 (Beta), B.1.1.28.1/P.1 (Gamma), B.1.617.2 (Delta), B.1.617.1 (Kappa), and B.1.1.529 (Omicron). These variants evolved in different regions and exhibited varying degrees of transmissibility, immune evasion, and disease severity [19,20,21,22,23,24]. The Omicron variant, first identified in South Africa in late 2021, rapidly spread worldwide, demonstrating a significantly higher transmission rate compared to the Delta variant and earlier SARS-CoV-2 lineages [24]. Omicron’s global impact was largely due to its 32-spike protein mutations, which is double the number found in the Delta variant, particularly in the spike (S) protein that plays a key role in viral entry and immune recognition [25]. Omicron has since become the dominant variant. The BA.1.1 and BA.2 lineages of the Omicron variant exhibit 57–59 mutations, which is 2 to 2.7 times more than those observed in the Alpha and Delta lineages [26]. This extensive genetic diversity has given rise to multiple subvariants and continues to drive the emergence of new lineages.

Previous studies have provided a critical understand into the genomic evolution and epidemiology of SARS-CoV-2 in Brazil. Giovanetti et al. (2022) [27] highlighted the co-circulation of multiple SARS-CoV-2 lineages and the emergence of key variants of concern (Gamma and Zeta) during the early pandemic, emphasizing the impact of unrestricted transmission. Menezes et al. (2022) [28] conducted a systematic review of genomic surveillance, documenting variant replacement patterns and the challenges in achieving comprehensive surveillance across Brazil. Lamarca et al. (2023) [29] investigated the introduction and spread of Omicron BA.1 and BA.2, identifying São Paulo as a critical dispersal hub and underscoring the need for targeted surveillance at entry points such as airports. Building on these foundational studies, this research extends the timeline to the post-peak pandemic period (2022–2024) and evaluates the evolution of variants within a new epidemiological landscape shaped by routine vaccination and the lifting of non-pharmacological interventions.

After the major crisis experienced between 2020 and 2021, Brazil has increased vaccination efforts, and most activities have returned to pre-pandemic levels following a marked decrease in cases and hospitalizations, notably from the second quarter of 2022. This study aims to characterize the genomic diversity of SARS-CoV-2 in Brazil from 1 July 2022 to 31 July 2024 using an extensive dataset of sequences available in the GISAID database. The specific objectives are as follows: (i) evaluate the relationship between the number of confirmed COVID-19 cases and sequencing efforts across various Brazilian states; (ii) analyze the regional distribution of genetic subvariants, with a particular focus on the frequency of single nucleotide variants (SNVs) across the five major regions of the country; (iii) identify and classify viral lineages using the Pangolin tool; and (iv) conduct phylogenetic analysis with a representative sample of the viral lineages. This research is critical for understanding genomic changes in the virus, offering data that can inform genomic surveillance strategies and enhance pandemic control efforts in Brazil.

## 2. Material and Methods

### 2.1. Data Retrieval

Complete SARS-CoV-2 whole-genome sequences from COVID-19 cases in Brazil were obtained from the Global Initiative on Sharing Avian Influenza Data-EpiCoV (GISAID-EpiCoV) platform (https://www.gisaid.org/, accessed on 9 August 2024) [30]. To be included in the analysis, genomes were required to exceed 29,000 base pairs (bps) in length and had to be submitted between 1 July 2022 and 31 July 2024. The SARS-CoV-2 reference genome, Wuhan-Hu-1, was also retrieved from the NCBI database (Accession Number: NC_045512.2).

### 2.2. Data Processing

To evaluate the relationship between COVID-19 cases and sequencing efforts across Brazilian states, data on the total number of confirmed cases and SARS-CoV-2 genomes sequenced were collected for each state. COVID-19 case data from 1 July 2022 to 31 July 2024 were obtained from publicly available databases maintained by the Brazilian Ministry of Health (https://covid.saude.gov.br/, accessed on 1 September 2024). The sequencing rate, defined as the number of genomes sequenced per million confirmed cases, was calculated by dividing the number of sequenced genomes by the total number of confirmed cases and then multiplying by 1,000,000. This metric facilitated standardized comparisons of sequencing efforts across states with varying caseloads. Pearson’s correlation coefficient was used to quantify the strength and direction of the association between the total number of cases and the number of genomes sequenced. All analyses, including the creation of scatter plots and bar graphs, were conducted using the R software version: 3.5.1 with the ggplot2 package [31].

Mutational analysis of SARS-CoV-2 genomes across Brazilian regions and the allele frequencies of single nucleotide variants (SNVs) were performed following established protocols [32]. The dataset was stratified into five Brazilian regions: Central-West, Northeast, North, South, and Southeast. Genomes were aligned to the SARS-CoV-2 reference genome using the Minimap2 aligner [33]. The resulting SAM files were sorted, converted to BAM format, and indexed using Samtools v1.9 (The Sanger Institute, Hinxton, UK) [34]. Variant calling was conducted with BCFtools using the mpileup and call functions to generate genomic VCF files, which were subsequently filtered with the BCFtools filter tool to produce the final VCF file. The variant effect predictor (VEP) was employed to assess the functional impacts of the identified subvariants on SARS-CoV-2 transcripts [35].

### 2.3. Pango Lineages and Phylogenetics Analysis

Pango lineages were assigned to each genome using Pangolin v4.3.1 (https://github.com/cov-lineages/pangolin, accessed on 29 August 2024) [36]. Phylogenetic analysis of the SARS-CoV-2 genomes from Brazil was performed using the Nextstrain platform (https://nextstrain.org/ncov, accessed on 25 September 2024) [37]. Due to the large volume of genomes sequenced and retrieved from GISAID, a representative subset of 13,416 genomes was selected using the subsample-max-sequence function in the Nextstrain workflow [37]. This selection ensured equitable representation across the Brazilian states, covering the period from 1 July 2022 to 31 July 2024. The subsampling process was stratified by geographic region (Brazilian states), year, and month to achieve a balanced spatial and temporal diversity through the group-by region year month option. After subsampling, the Nextstrain workflow was executed, encompassing alignment, maximum-likelihood phylodynamic analysis, the temporal dating of ancestral nodes, discrete trait geographic reconstruction, and the visualization of the phylogenetic results.

## 3. Results

**Figure 1:**
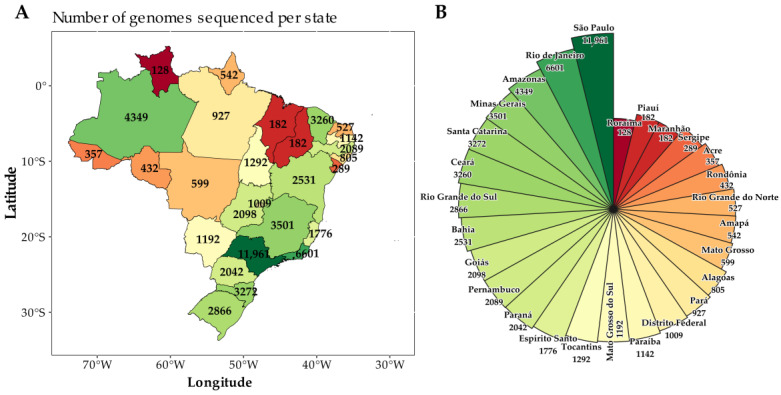
(**A**) Map of Brazil showing the distribution of 55,951 SARS-CoV-2 genomes sequenced by state from 1 July 2022 to 31 July 2024. (**B**) Pie chart illustrating the number of genomes sequenced per state during the same period. The colors of the states on the map correspond to the same colors in the pie chart.

In this study, 55,951 SARS-CoV-2 sequences collected between 1 July 2022 and 31 July 2024 were analyzed to illustrate the distribution of sequenced genomes across the Brazilian states during the study period. A higher concentration of sequences was observed in the states of São Paulo (*N* = 11,961; 21.4%), Rio de Janeiro (*N* = 6601; 11.8%), Amazonas (*N* = 4349; 7.8%), Minas Gerais (*N* = 3501; 6,3%), Santa Catarina (*N* = 3272; 5.8%), Ceará (*N* = 3260; 5.8%), and Rio Grande do Sul (*N* = 2866; 5.1%) (Figure 1A,B).

The sequencing efforts across the Brazilian states in response to the COVID-19 pandemic showed significant variability compared to the total number of confirmed cases. A standardized comparison across states with different epidemic burdens was achieved by calculating the sequencing rate—defined as the number of genomes sequenced per million cases. States like São Paulo and Rio de Janeiro, which recorded the highest case counts, also conducted the largest absolute number of genome sequences. However, their sequencing rates were moderate when normalized to per million cases (Table S1). In contrast, states such as Amapá, Amazonas, Ceará, and Tocantins, which had relatively low to moderate case counts, demonstrated higher sequencing efforts. This suggests that these states placed a significant focus on genomic surveillance, prioritizing sequencing despite having fewer cases (Table S1). Conversely, states like Maranhão, Piauí, and Roraima exhibited lower sequencing rates in relation to their case counts. A correlation analysis between the total number of confirmed cases and sequencing efforts yielded a Pearson correlation coefficient of 0.79, indicating a strong positive relationship (Supplementary Figure S1). This suggests that states with more cases generally sequenced more genomes, though some states displayed either disproportionately high or low sequencing efforts relative to their case burden.

This underscores the uneven distribution of genomic surveillance across Brazil, with certain states allocating more resources to sequencing efforts regardless of their epidemic burden. These findings highlight disparities in state-level priorities and emphasize the essential role of collaborative networks and funding in bolstering nationwide genomic surveillance. Key institutions, including the Oswaldo Cruz Foundation (FIOCRUZ), Instituto Butantan, and the Central Public Health Laboratories, in partnership with initiatives like the Corona-Ômica Network, have played a pivotal role in developing and implementing sequencing strategies. These collaborative efforts have been instrumental in strengthening technical capacities, facilitating data sharing, and prioritizing genomic sequencing in resource-constrained states, thereby significantly enhancing Brazil’s overall genomic surveillance system.

**Figure 2:**
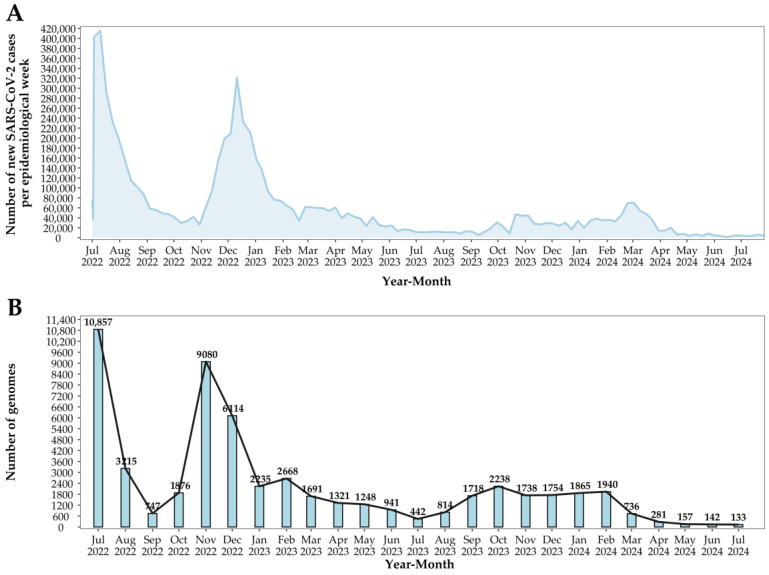
Epidemiology of SARS-CoV-2 in Brazil. (**A**) Number of new SARS-CoV-2 cases by epidemiological week from July 2022 to July 2024. (**B**) Number of SARS-CoV-2 genomes sequenced monthly from July 2022 to June 2024.

Between 1 July 2022, and 31 December 2022, a total of 31,889 SARS-CoV-2 genomes were sequenced. In 2023, this number decreased to 18,808, and from 1 January 2024 to 31 July 2024, only 5254 genomes were sequenced. These data indicate a marked decline in both COVID-19 cases and SARS-CoV-2 genomic surveillance in Brazil over this period (Figure 2A). In July 2022, Brazil reported 1,653,155 new COVID-19 cases, with 10,857 viral genomes sequenced. Subsequently, there was a steady decrease in case numbers, with some fluctuations, particularly in November and December 2022, likely driven by seasonal factors and holiday gatherings. For instance, in December 2022, 973,494 cases were reported, along with 6114 genomes sequenced. Starting from January 2023, the decline in both cases and genomic sequencing became more pronounced, reaching a low in July 2024, where only 27,397 new cases and 133 genomes were sequenced (Figure 2B).

### 3.1. Pango Lineages

**Figure 3:**
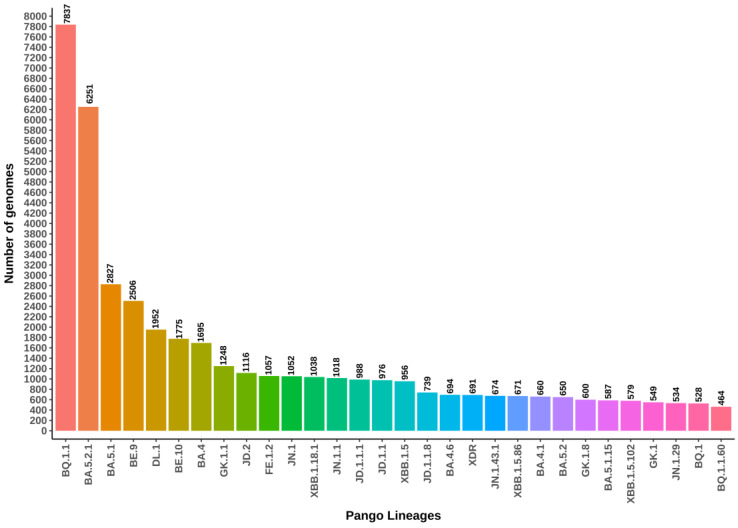
The top thirty SARS-CoV-2 lineages circulating in Brazil, identified from genomes submitted to the GISAID repository between 1 July 2022 and 31 July 2024. Colors in the heatmap corresponds to a Pango Lineages, with red indicating the highest number of genomes and pink the lowest. Intermediate values are represented by a gradient transitioning through green and blue, reflecting a linear scale of genome counts.

Between 1 July 2022 and 31 July 2024, a total of 626 distinct SARS-CoV-2 lineages were identified to be circulating in Brazil. The majority of the genomes deposited in the GISAID database and analyzed in this study belong to the following lineages: BQ.1.1 (*N* = 1850; 14.01%), BA.5.2.1 (*N* = 6251; 11.17%), BA.5.1 (*N* = 2827; 5.05%), BE.9 (*N* = 2506; 4.48%), DL.1 (*N* = 1952; 3.49%), BE.10 (*N* = 1775; 3.17%), BA.4 (*N* = 1695; 3.03%), GK.1.1 (*N* = 1248; 2.23%), JD.2 (*N* = 1116; 1.99%), and FE.1.2 (*N* = 1057; 1.89%) (Figure 3). These 10 lineages account for approximately 71.95% of all sequenced genomes in Brazil. Notably, 179 lineages (28.6% of the total) were represented by a single genome within the database. Figure 3 illustrates the 30 most frequently identified lineages.

When analyzing annual trends, 258 lineages were identified in 2022, with the most prevalent being BQ.1.1 (*N* = 6708; 21.0% of the total genomes sequenced that year), followed by BA.5.2.1 (*N* = 6238; 19.6%), BA.5.1 (*N* = 2826; 8.9%), BE.9 (*N* = 2260; 7.1%), and DL.1 (*N* = 1897; 5.9%) (Supplementary Figure S2). In 2023, the number of circulating lineages increased to 370, with the most frequent being GK.1.1 (*N* = 1244; 6.61%), BQ.1.1 (*N* = 1128; 6.0%), JD.2 (*N* = 1101; 5.85%), FE.1.2 (*N* = 1057; 5.62%), and XBB.1.18.1 (*N* = 1025; 5.45%) (Supplementary Figure S3). By 2024 (up to July), 150 lineages had been identified, with JN.1 (*N* = 953; 18.1%), XDR (*N* = 645; 12.3%), JN.1.1 (*N* = 570; 10.8%), JN.1.43.1 (*N* = 438; 8.3%), and JN.1.29 (*N* = 305; 5.8%) being the most prevalent (Supplementary Figure S4).

**Figure 4:**
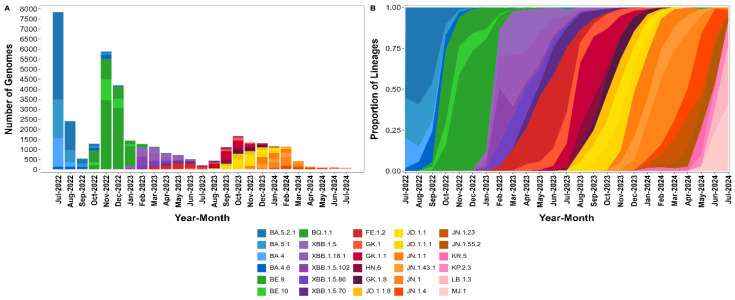
Distribution of SARS-CoV-2 lineages in this study from July 2022 to July 2024. (**A**) Absolute number of SARS-CoV-2 genomes for the top three most frequent sublineages per month, illustrating the dominance and transition of sublineages over time. (**B**) Relative frequency of these sublineages, emphasizing the progressive shifts in viral dominance.

We also performed a month-by-month analysis of the SARS-CoV-2 lineages, focusing on the most prevalent lineages each month (Figure 4A,B). A total of 37,171 SARS-CoV-2 genomes, representing 66.4% of the entire dataset, were analyzed. The 2022 monthly analysis revealed a sustained dominance of the BA.5 and BA.4 sublineages over several months. In July, the most frequently detected sublineages were BA.5.2.1 (4331 occurrences), BA.5.1 (1931 occurrences), and BA.4 (1429 occurrences). This pattern persisted in August, with BA.5.2.1 (1426 occurrences), BA.5.1 (623 occurrences), and BA.4 (219 occurrences) continuing to dominate. By September, BA.5.2.1 remained the most frequent sublineage (246 occurrences), followed by BA.5.1 (124 occurrences) and BA.4.6 (100 occurrences), indicating the sustained dominance of BA.5 and its sublineages through the latter half of the year (Figure 4A,B).

In 2023, a shift in the circulating sublineages was observed, with XBB recombinant lineages becoming increasingly prominent (Figure 4A,B). In January, BQ.1.1 was the most frequent sublineage, with 989 occurrences, followed by XBB.1.5 (66 occurrences) and XBB.1.18.1 (61 occurrences). By February, XBB.1.5.102 emerged as the most frequent sublineage with 428 occurrences, followed by XBB.1.18.1 (242 occurrences) and XBB.1.5 (209 occurrences). This trend continued into March, where XBB.1.18.1 (354 occurrences) led, followed by XBB.1.5 (295 occurrences) and XBB.1.5.86 (224 occurrences). Throughout mid-2023, XBB-related sublineages remained prevalent, with XBB.1.5 and its subvariants accounting for a significant proportion of cases. However, new subvariants continued to emerge and circulate. By the latter half of the year, beginning in July, the GK.1.1 sublineage began to rise in prevalence, with 170 occurrences in July, peaking at 413 in September 2023 and making it the most dominant lineage during this period. Other subvariants like JD.1.1.8, also showed a substantial increase, particularly in October 2023 (268 occurrences). By the end of 2023, JD.1.1 and JD.1.1.1 became more prominent, with 298 and 277 occurrences, respectively, in November. This rise in JD sublineages was accompanied by a decline in the previously dominant XBB-related sublineages, marking another shift in the viral landscape (Figure 4A,B).

In 2024, the SARS-CoV-2 landscape in Brazil continued to evolve, with several new sublineages emerging as dominant variants (Figure 4A,B). The year began with the JN.1 sublineage and its subvariants leading the circulation. In January 2024, JN.1 was the most prevalent sublineage with 311 occurrences, followed closely by JN.1.1 (270 occurrences) and JN.1.43.1 (155 occurrences). The XDR sublineage also showed a significant circulation early in the year, with 251 occurrences. As the year progressed, JN.1 remained dominant until March 2024, where its prevalence began to taper, recording 174 occurrences, while JN.1.43.1 (58 occurrences) and JN.1.4 (47 occurrences) persisted. Interestingly, the XDR subvariant also declined, with 99 occurrences in March, indicating a potential shift in subvariant dominance. By May and June 2024, new sublineages such as KP.2.3 and LB.1.3 started to emerge, with KP.2.3 reaching 12 occurrences and MJ.1 recording 23 occurrences in June. The overall trend in 2024 suggested a gradual transition away from the dominance of the JN.1 sublineage, with newer subvariants slowly gaining prevalence as the year progressed (Figure 4A,B).

### 3.2. An Analysis of SARS-CoV-2 Mutations Across Brazilian Regions

**Figure 5:**
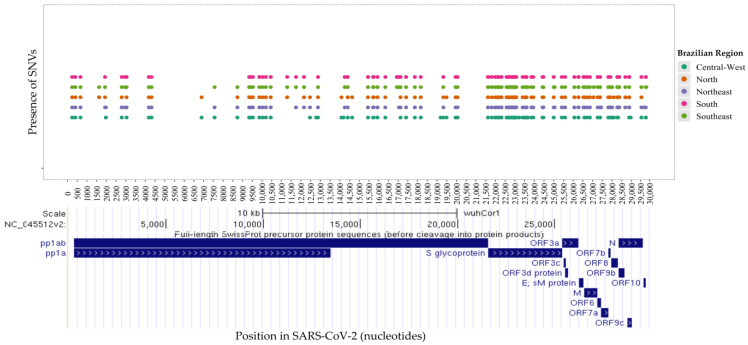
Distribution of all single nucleotide variants identified across regions along the nucleotide sequence positions of SARS-CoV-2. The coding regions (ORFs) of SARS-CoV-2 are depicted at the bottom.

In this study, we analyzed the distribution of mutations in 55,951 Brazilian SARS-CoV-2 sequences obtained from the public GISAID repository between 1 July 2022 and 31 July 2024. Our analysis identified single nucleotide variants (SNVs) at 148 distinct positions in the viral genome (Figure 5). Of these, 91 SNVs (61.5%) were shared across all five Brazilian regions (Figure 5). The highest number of SNVs was recorded in the Southeast (125 SNVs), followed closely by the South (116 SNVs), North (116 SNVs), Northeast (116 SNVs), and Central-West (115 SNVs) regions.

Notably, six SNVs were unique to the Central-West region (C12789T-AF = 22.3%, C14189T-AF = 43.8%, C19185T-AF = 23.8%, C22295A-AF = 19.4%, G23222A-AF = 21.2%, C28958A-AF = 16.5%). Two region-specific SNVs were identified in the Northeast region (T27438C-AF = 23.0% and C29666T-AF = 23.1%), and two in the North region (A27038G-AF = 35.9% and T28693-AF = 23.6%). The Southeast region exhibited four unique SNVs (C27059T-AF = 24.0%, A28330G-AF = 20.8%, G29757T-AF = 91.5%, and G29759C-AF = 79.1%), while four SNVs were also identified in the South region (T21633C-AF = 33.8%, C21635T-AF = 36.5%, C21636A-AF = 34.9%, and C29738G-AF = 29.2%).

**Figure 6:**
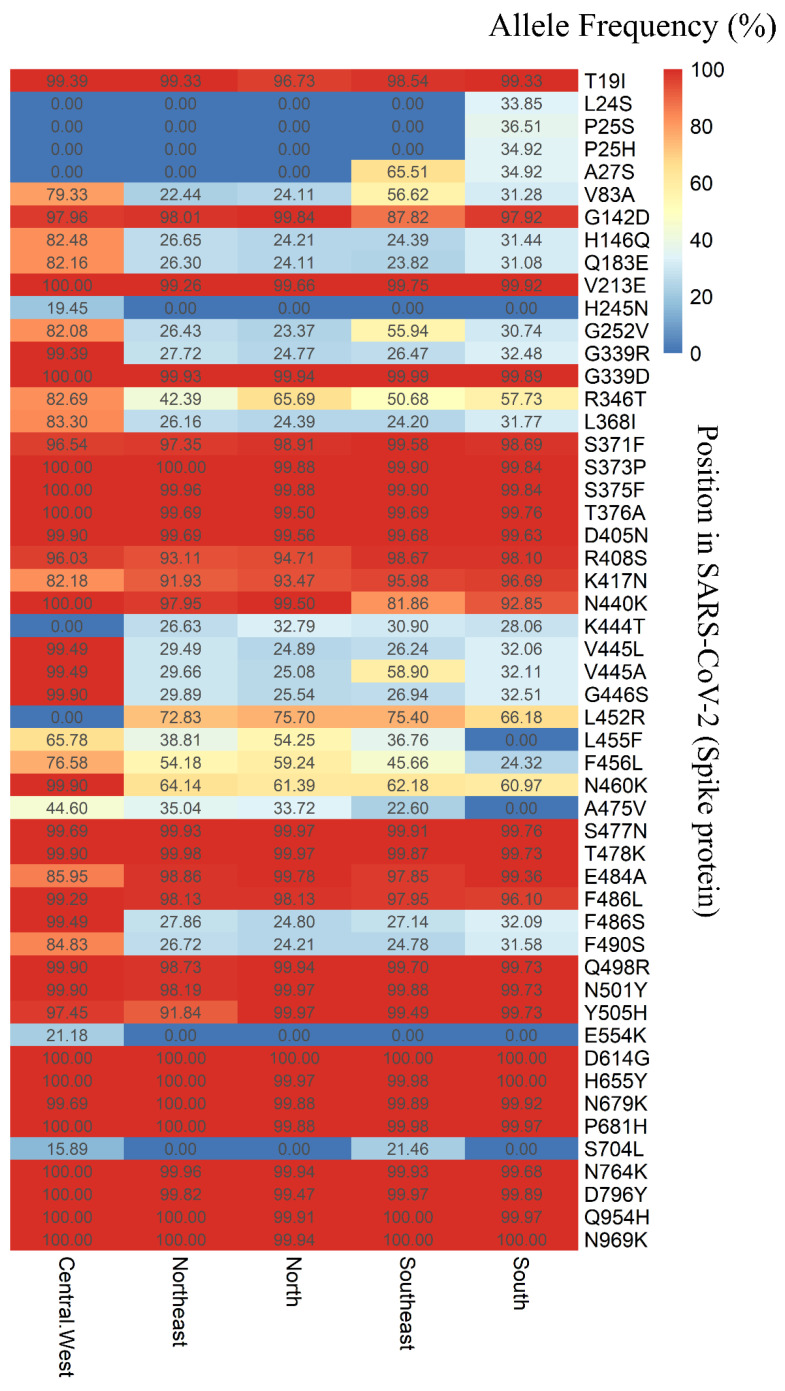
Allele frequency (expressed as a percentage) of variants in the spike protein of Brazilian SARS-CoV-2 sequences, based on data available from the GISAID-EpiCoV platform, spanning July 2022 to July 2024.

Among the 148 SNVs, 139 were located in the coding regions of the viral genome, one in the 5′ UTR (C241T), five in the 3′ UTR (G29734T, G29736C, G29737A, C29738G, G29757T, and G29759C), one upstream of ORF8 (C27889T), and one upstream of the N gene (A28271T). Of the 139 mutations in the coding region, 42 were synonymous (silent), while 96 were predicted to result in amino acid substitutions (missense), including a stop codon in the ORF8 gene (G27915T). Notably, fifty-five mutations were identified in the spike (S) gene, comprising three synonymous and fifty-two missense mutations. Figure 6 illustrates the allele frequencies of the SNVs in the S gene across the five Brazilian regions.

The SNVs H245N (C22295A) and E554K (G23222A) were exclusively identified in the Central-West region, with allele frequencies of 19.45% and 21.18%, respectively (Figure 6). In the South region, SNVs L24S (T21633C), P25S (C21635T), and P25H (C21636A) were observed, with allele frequencies of 33.85%, 36.51%, and 34.92%, respectively. The SNV A27S (G21641T) was detected exclusively in the Southeast and South regions, showing allele frequencies of 30.90% and 34.92%, respectively. Additionally, the SNV S704L (C23673T) was identified in both the Central-West and Southeast regions, with allele frequencies of 15.89% and 21.46%, respectively (Figure 6).

Among the 139 SNVs located in the coding regions of the viral genome, ORF1ab, which constitutes approximately 67% of the genome and encodes 16 non-structural proteins, exhibited 49 substitution mutations, comprising 25 missense and 24 synonymous mutations. Notably, the spike protein harbored fifty-five mutations, of which fifty-two (94.5%) were missense and three (5.5%) were synonymous. Additionally, we identified a deletion at position 27,915 (Gga/Tga), resulting in a stop codon in ORF8 (Supplementary Figure S5).

### 3.3. Phylogenetic Analysis

**Figure 7:**
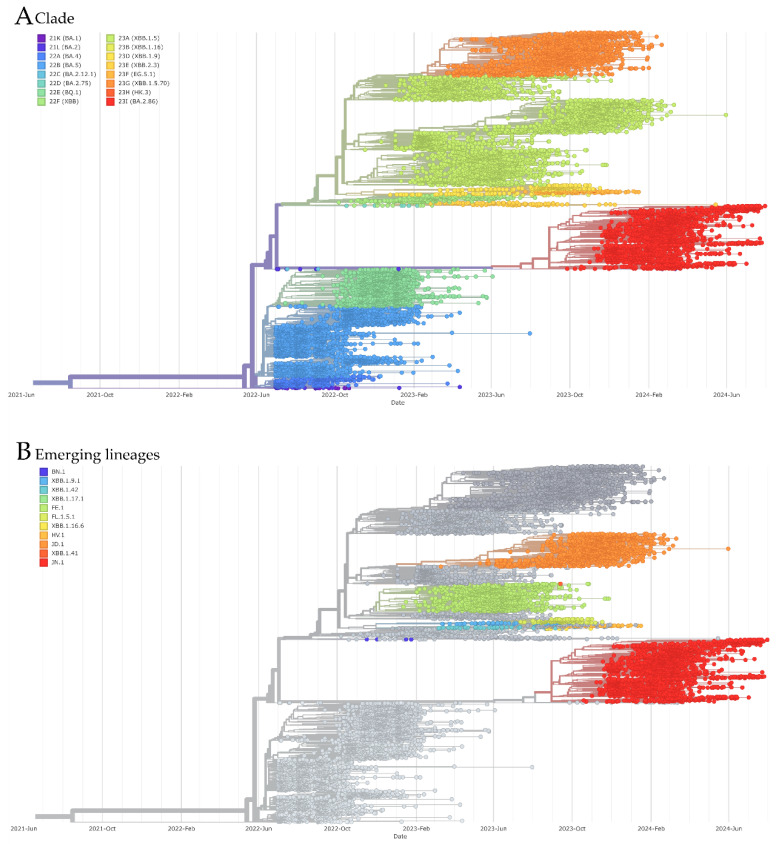
Phylogenetic tree and clade assignment for 13,416 Brazilian SARS-CoV-2 genomes. (**A**) Phylogenetic tree colored according to Nextstrain clades. (**B**) Phylogenetic tree colored according to emerging lineages.

A total of 13,416 Brazilian genomes, representing all states, were included in the phylogenetic analysis conducted using the Nextstrain pipeline (Figure 7A). These genomes were classified into 16 distinct clades. In 2022, lineages from 10 clades (21K, 21L, 22A, 22B, 22C, 22D, 22E, 22F, 23A, and 23D) were circulating, with clade 22B being the most predominant, followed by 22A. In 2023, new clades emerged (23B, 23E, 23F, 23G, 23H, and 23I), with clade 23A becoming the most prevalent, peaking in March and April. Clades such as 23G and 22E also exhibited significant activity. By the end of 2023, clade 23I (BA.2.86) emerged, representing the majority of sequences in 2024 and signaling a major shift in the viral landscape. This progression underscores the ongoing evolution of SARS-CoV-2, with different variants dominating at different times. Additionally, eleven lineages were classified as emerging (BN.1, XBB.1.9.1, XBB.1.42, XBB.1.17.1, FE.1, FE.1.5.1, XBB.1.16.6, HV.1, JD.1, XBB.1.41, and JN.1), with JN.1, FE.1, and JD.1 showing particularly high frequencies (Figure 7B).

## 4. Discussion

This study presents findings from a molecular epidemiological analysis of 55,951 SARS-CoV-2 sequences collected from the GISAID database between 1 July 2022 and 31 July 2024 in Brazil. The results highlight the key aspects of genomic surveillance efforts and the evolving viral landscape. A critical observation is the significant variability in sequencing efforts across Brazilian states, despite the uniformly high public health burden of COVID-19. For example, states such as São Paulo and Rio de Janeiro reported a high number of COVID-19 cases and genome sequences; however, their sequencing rates per million cases were moderate. In contrast, states with fewer cases, such as Tocantins, Amapá, Amazonas, and Ceará, exhibited disproportionately high sequencing rates. This discrepancy, particularly evident in states like Maranhão, Piauí, and Roraima, may reflect heterogeneity in resource allocation, potentially hindering the effective monitoring of emerging variants. This imbalance in SARS-CoV-2 genomic surveillance has also been observed globally, driven by socioeconomic factors and pre-pandemic laboratory and surveillance capacities [38].

Genomic sequencing has been pivotal in tracking the evolution and spread of SARS-CoV-2 globally, providing essential information to support public health initiatives [23,39,40,41,42]. In Brazil, a nation that faced significant challenges during the COVID-19 pandemic, research has demonstrated how the timing and implementation of restriction measures influenced viral transmission by integrating genomic and epidemiological data [27]. A key finding from these studies was the identification of multiple independent introductions of SARS-CoV-2 into Brazil, primarily from Europe, during the early stages of the pandemic [27]. As the situation evolved, Brazil transitioned from being a major importer of the virus to a significant exporter, particularly as variants of concern (VOCs) and variants under monitoring (VUMs) began to emerge [27]. The continuous emergence of new variants underscores the critical need for robust genomic surveillance, as reductions in sequencing efforts risk delaying the detection of potentially concerning lineages. This situation emphasizes the importance of generating, reporting, sharing, and making sequencing data publicly available to facilitate real-time global surveillance and informs response strategies against evolving threats. Notably, regions and states with laboratories or research institutions possessing greater expertise and funding for genomic surveillance achieved higher rates of sequenced genomes relative to total cases. This finding highlights the importance of financing and capacity building to effectively monitor the evolution of emerging virus—a strategic priority that should not be overlooked by governments and society.

Our results indicate that the evolution of SARS-CoV-2 in Brazil has been marked by the shifting dominance of various lineages over time. In 2022, the BA.5 and BA.4 sublineages were the most prevalent, with BA.5.2.1 and BA.5.1 maintaining high frequencies throughout the year. By 2023, however, the XBB recombinant lineages emerged as more dominant subvariants. The XBB lineage was first identified in October 2022, initially accounting for less than 10% of the circulating lineages [43]. Between January and February 2023, these variants rapidly increased in prevalence, particularly those harboring the F486P mutation. This rise was driven by the gradual accumulation of mutations in the receptor-binding domain (RBD) of the spike protein, including F486P, F456L, and L455F, which significantly enhanced the virus’s ability to evade immunity and bind more effectively to the ACE2 receptor [43,44].

In August 2023, a sublineage designated as BA.2.86 was identified in multiple countries, differentiated from its parent BA.2 lineage by more than 30 mutations in the spike protein. The BA.2.86 sublineage subsequently evolved rapidly, giving rise to the JN.1 sublineage, which emerged in September 2023. This sublineage quickly became predominant globally and was the primary variant driving the epidemic surge observed in December 2023 and January 2024 [45]. In response to its increasing prevalence, the World Health Organization classified JN.1 as a variant of interest [46]. In our study, JN.1 emerged as the dominant lineage in 2024, alongside newly identified lineages such as XDR, KP.2.3, and LB.1.3. This evolution underscores the ongoing adaptation and recombination of the virus as it continues to spread within the population, contributing to a highly dynamic viral landscape.

The high frequency of mutations in the spike protein, which is crucial for viral entry into host cells and serves as a primary target for vaccines, underscores the importance of continuously evaluating vaccination strategies. For example, mutations such as N501Y and Q498R enhance the virus’s transmissibility by increasing the binding affinity of the receptor-binding domain (RBD) for the ACE-2 receptor, thereby facilitating viral entry. Additionally, mutations H655Y and N679K, located near the furin cleavage site, promote enhanced cleavage at the S1/S2 junction. The P681H mutation further increases S1/S2 cleavage, contributing to the virus’s enhanced ability to spread [47].

The phylogenetic analysis highlights the complexity of SARS-CoV-2 evolution in Brazil, revealing the dynamic circulation of variants over time. In 2022, clade 22B predominated, reflecting the continued transmission of its associated subvariants. However, the emergence of new clades in 2023, such as 23A and 23I, demonstrates the virus’s adaptation to immune pressures and genetic variability within the population. The rise in clade 23I (BA.2.86) in early 2024, with its significant representation among the analyzed genomes, marks a critical shift in the viral landscape, underscoring the ongoing evolution and diversification of the virus. The identification of multiple emerging lineages, including JN.1 and FE.1, emphasizes the importance of sustained genomic surveillance to monitor viral evolution and inform public health strategies, particularly in a context where the emergence of new variants could impact the effectiveness of existing interventions.

While this study provides valuable information about SARS-CoV-2 evolution and variant dynamics in Brazil over the past two years, several limitations should be acknowledged. The relatively small number of sequenced genomes may not fully capture the diversity of circulating variants, particularly given the disparities in sequencing efforts across states. Some regions exhibited a significant lack of genomic surveillance, potentially hindering the effective monitoring of ongoing viral transmission, especially as a large portion of the population becomes immunized. Furthermore, the uneven distribution of sequenced genomes among states underscores the need for more equitable resource allocation to strengthen genomic surveillance efforts. Despite these limitations, this analysis utilizes data from the GISAID database to provide a comprehensive overview of the COVID-19 pandemic in Brazil, highlighting the critical importance of sustained genomic monitoring as we navigate the evolving landscape of the virus.

## 5. Conclusions

In conclusion, this study underscores the dynamic nature of SARS-CoV-2 in Brazil from July 2022 to July 2024, emphasizing the importance of genomic surveillance for understanding viral evolution. The analysis of 55,951 sequences revealed significant regional disparities in sequencing efforts and the circulation of distinct lineages, highlighting the need for more equitable resource allocation across states. Our findings indicate a notable shift in dominant subvariants, from BA.5 in 2022 to XBB and JN.1 in the following years, reflecting the virus’s ongoing adaptation. These insights emphasize the critical role of continuous genomic monitoring in tracking the emergence of new variants and informing effective public health strategies. As Brazil navigates the complexities of COVID-19, the lessons learned from this analysis will be invaluable in guiding future efforts to mitigate the virus’s impact and safeguard public health.

## Data Availability

The authors declare that all data supporting the findings of this study are available within the paper. The SARS-CoV-2 genomes were obtained from the GISAID database and were described in Table S2.

## Supplementary Materials

The following supporting information can be downloaded at the following website: https://www.mdpi.com/article/10.3390/v17010064/s1, Table S1. Sequencing efforts compared to COVID-19 cases by Brazilian states. Table S2. GISAID data availability. Figure S1. Correlation between COVID-19 cases and sequencing efforts in Brazilian states. Figure S2. The top thirty SARS-CoV-2 lineages circulating in Brazil in 2022. Figure S3. The top thirty SARS-CoV-2 lineages circulating in Brazil in 2023. Figure S4. The top thirty SARS-CoV-2 lineages circulating in Brazil in 2024. Figure S5. Distribution of synonymous, missense, and stop mutations across genes and open reading frames.
