# Supplementary material for: Two Years of SARS-CoV-2 Omicron Genomic Evolution in Brazil (2022–2024): Subvariant Tracking and Assessment of Regional Sequencing Efforts
Source: Viruses. 2025 Jan 4;17(1):64. doi: 10.3390/v17010064 (PMC11768930; doi:10.3390/v17010064)
Supplement: Supplementary file 1 [file viruses-17-00064-s001.zip › viruses-3388077 - Supplementary_material.pdf]

**Table S1.** Sequencing Efforts Compared to COVID-19 Cases by Brazilian States. This table presents the total number of confirmed COVID-19 cases and the number of SARS-CoV-2 genomes sequenced for each Brazilian state. The sequencing rate is calculated as the number of genomes sequenced per million confirmed cases, facilitating a standardized comparison of sequencing efforts across states with differing epidemic burdens. States are listed alphabetically, with data covering the period from July 1, 2022, to July 31, 2024.

| Brazilian State | Sequencing | Cases | Sequencing Rate |
|---|---|---|---|
| Acre | 357 | 162,164 | 2201.48 |
| Alagoas | 805 | 212,814 | 3782.65 |
| Amapá | 542 | 129,574 | 4182.94 |
| Amazonas | 4349 | 419,544 | 10,366.02 |
| Bahia | 2531 | 1,142,856 | 2214.63 |
| Ceará | 3260 | 1,103,012 | 2955.54 |
| Espírito Santo | 1776 | 1,507,148 | 1178.38 |
| Federal District | 1009 | 861,548 | 1171.15 |
| Goiás | 2098 | 2,195,516 | 955.58 |
| Maranhão | 182 | 260,940 | 697.48 |
| Mato Grosso | 599 | 731,368 | 819.01 |
| Mato Grosso do Sul | 1192 | 508,484 | 2344.22 |
| Minas Gerais | 3501 | 4,185,624 | 836.43 |
| Pará | 927 | 550,404 | 1684.22 |
| Paraíba | 1142 | 522,566 | 2185.37 |
| Paraná | 2042 | 2,853,272 | 715.67 |
| Pernambuco | 2089 | 1,178,204 | 1773.04 |
| Piauí | 182 | 208,312 | 873.69 |
| Rio de Janeiro | 6601 | 3,203,600 | 2060.49 |
| Rio Grande do Norte | 527 | 428,164 | 1230.84 |
| Rio Grande do Sul | 2866 | 3,264,902 | 877.82 |
| Rondônia | 432 | 440,200 | 981.37 |
| Roraima | 128 | 122,220 | 1047.29 |
| Santa Catarina | 3272 | 1,683,250 | 1943.86 |
| São Paulo | 11,961 | 4,845,402 | 2468.53 |
| Sergipe | 289 | 179,000 | 1614.53 |
| Tocantins | 1292 | 292,600 | 4415.58 |

**Table S2. GISAID data availability.**

| Data | Description |
|---|---|
| GISAID Identifier | EPI_SET_241230zd |
| Doi link | 10.55876/gis8.241230zd |

**Observations:**

1) All genome sequences and associated metadata in this dataset are published in GISAID's EpiCoV database. To view the contributors of each individual sequence with details such as accession number, Virus name, Collection date, Originating Lab and Submitting Lab and the list of Authors, visit 10.55876/gis8.241230zd
2) Data Snapshot:
   EPI_SET_241230zd is composed of 55,951 individual genome sequences.
   The collection dates range from 2022-07-01 to 2024-07-31;
   Data were collected in 1 countries and territories;
   All sequences in this dataset are compared relative to hCoV-19/Wuhan/WIV04/2019 (WIV04), the official reference sequence employed by GISAID (EPI_ISL_402124).
   Learn more at https://gisaid.org/WIV04.

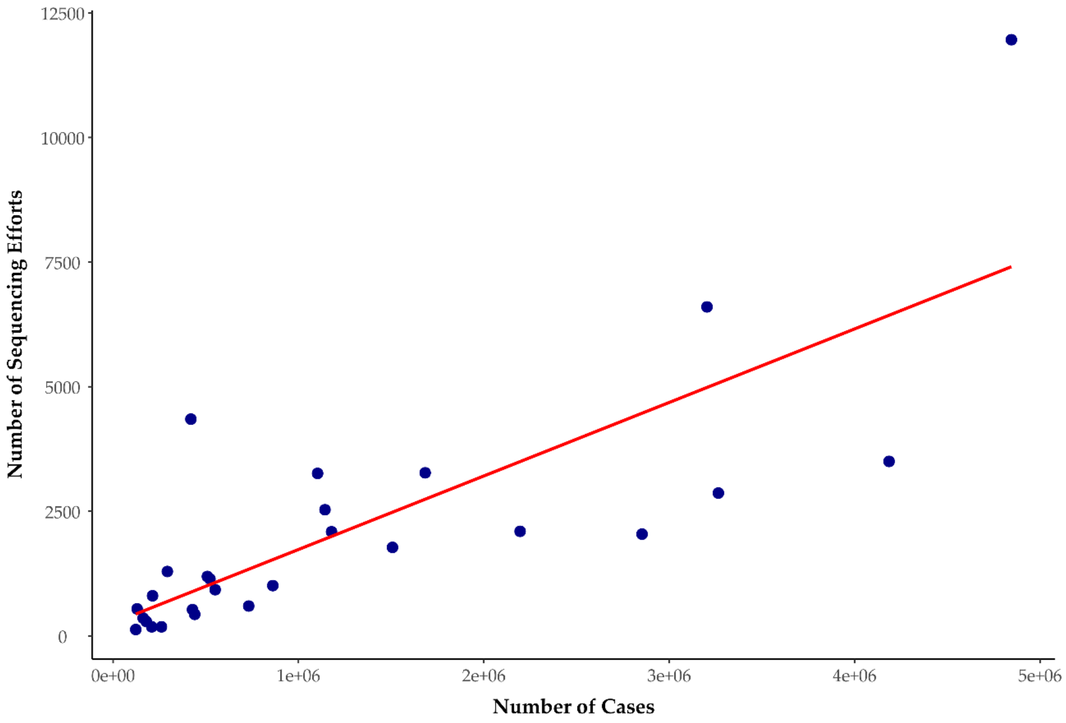


**Figure S1.** Correlation between COVID-19 Cases and Sequencing Efforts in Brazilian States. This scatter plot illustrates the relationship between the number of confirmed COVID-19 cases and the number of SARS-CoV-2 genomes sequenced across Brazilian states. Each point represents an individual state, with the x-axis depicting the number of confirmed cases and the y-axis representing the sequencing efforts. A red dashed line indicates the linear trend, fitted using linear regression, which highlights the positive correlation between the two variables. A Pearson correlation coefficient of 0.79 signifies a strong positive relationship, suggesting that states with higher case counts generally conducted more sequencing. However, the plot also demonstrates variability, as some states sequenced more or fewer genomes than expected based on their case numbers.

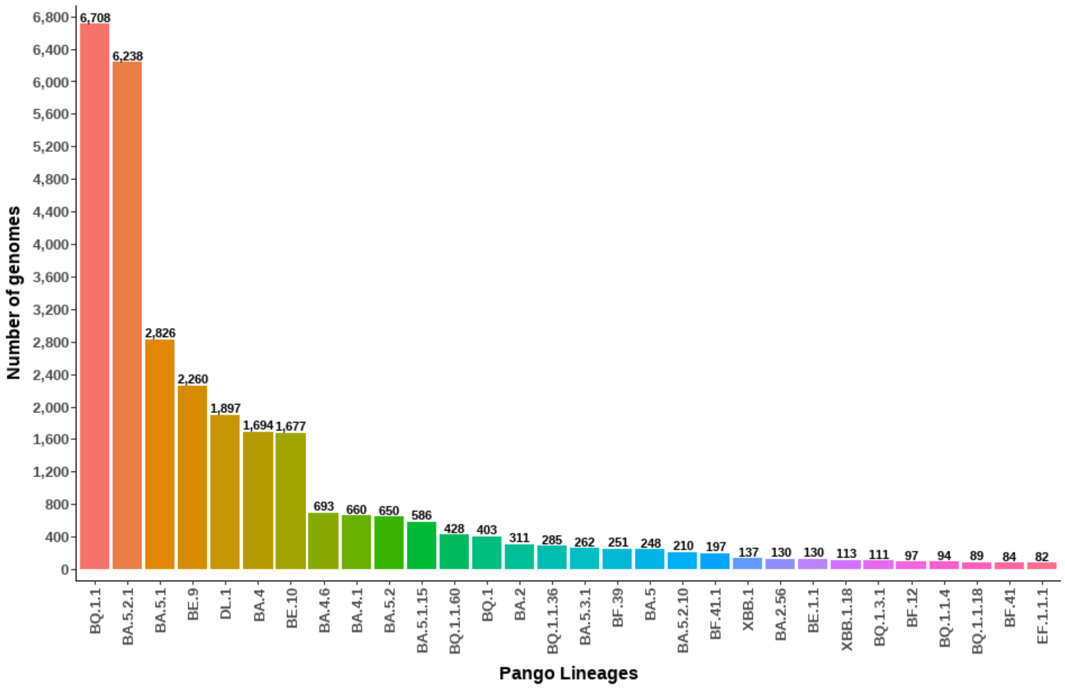


**Figure S2.** The top thirty SARS-CoV-2 lineages circulating in Brazil in 2022. The thirty most prevalent SARS-CoV-2 lineages identified from genomes submitted to the GISAID repository between July 1, 2022, and December 31, 2022.

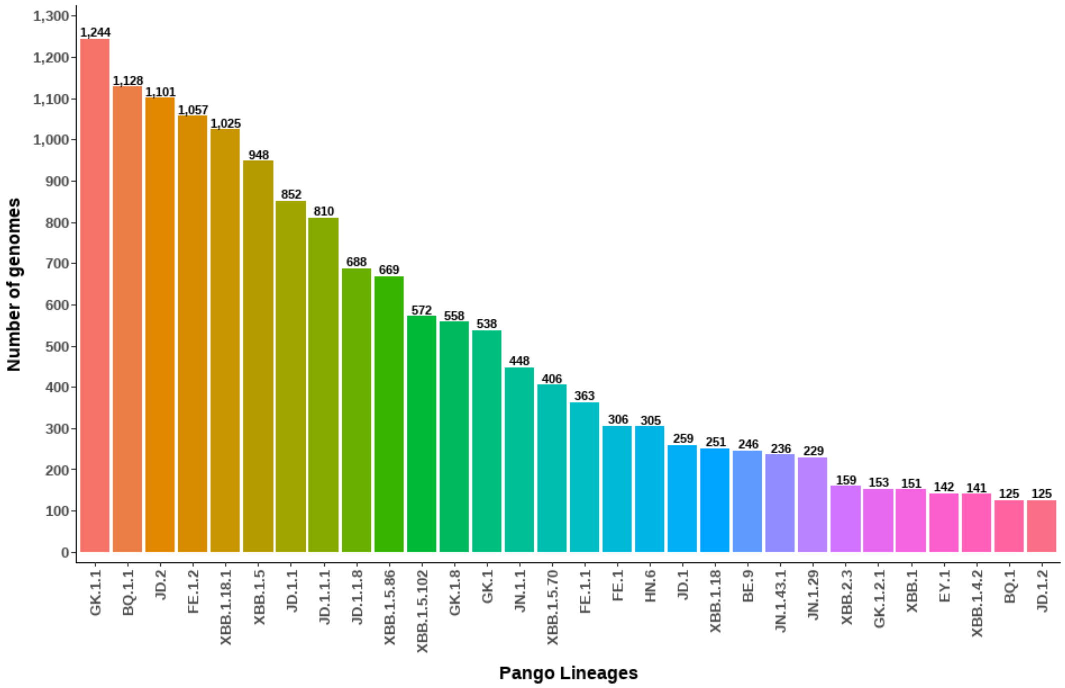


**Figure S3**. The top thirty SARS-CoV-2 lineages circulating in Brazin in 2023. The thirty most prevalent SARS-CoV-2 lineages identified from genomes submitted to the GISAID repository between January 1, 2023, and December 31, 2023.

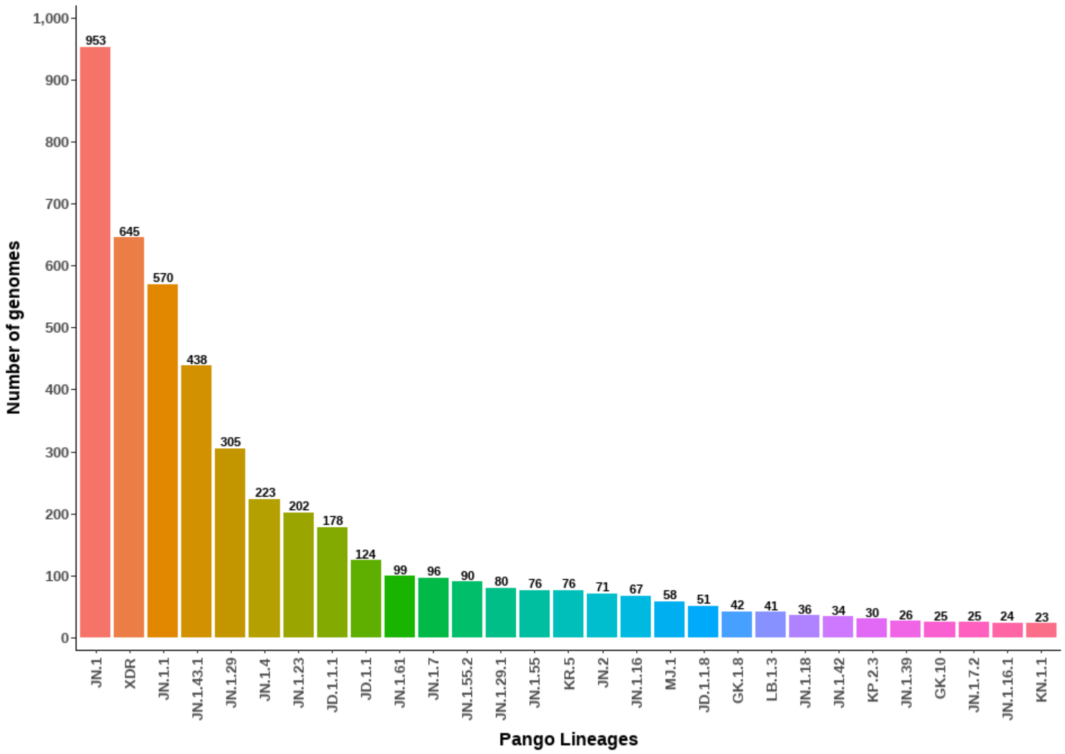


**Figure S4**. The top thirty SARS-CoV-2 lineages circulating in Brazil in 2024. The thirty most prevalent SARS-CoV-2 lineages identified from genomes submitted to the GISAID repository between January 1, 2024, and July 31, 2024.

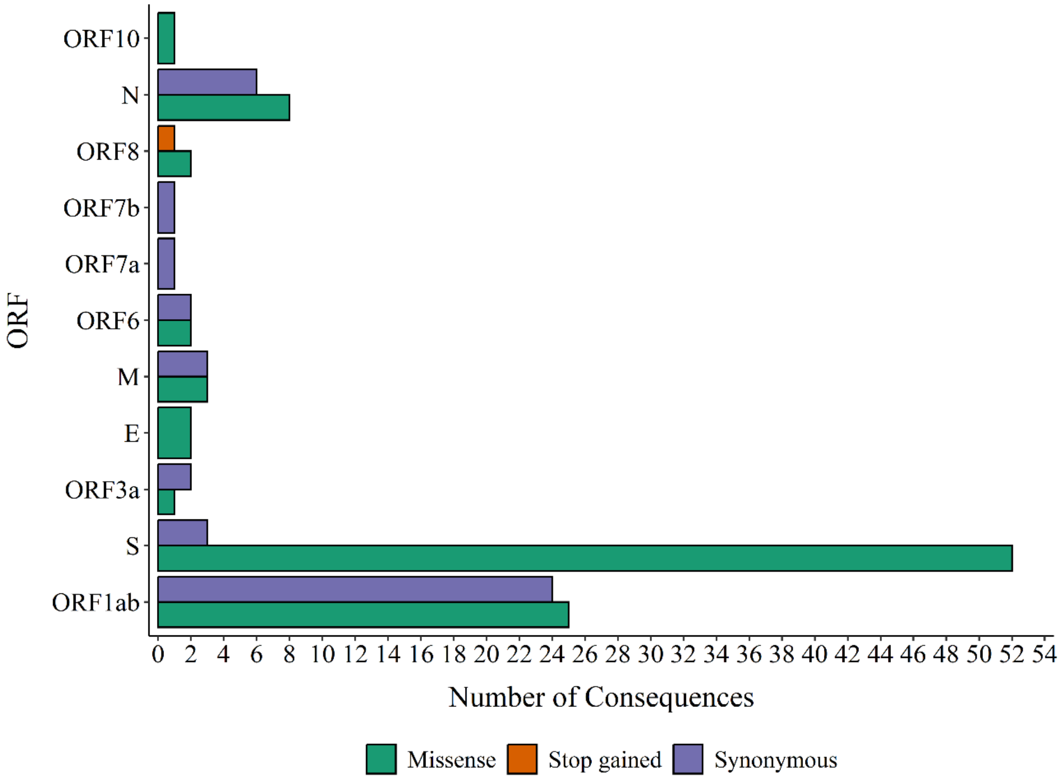


**Figure S5.** Distribution of synonymous, missense, and stop mutations across genes and open reading frames. The categorization of mutations identified using variant effect prediction programs, highlighting the frequency of synonymous, missense, and stop codon mutations in each gene or ORF of the SARS-CoV-2 genomes.
